# ‘If I were thirteen stone I’d be happier’: A narrative analysis of one woman’s story of decades of weight-cycling and dieting

**DOI:** 10.1177/13591053261417785

**Published:** 2026-02-18

**Authors:** Maxine Woolhouse, Irmgard Tischner

**Affiliations:** 1 Leeds Beckett University, UK; 2 Technische Hochschule Deggendorf, Germany

**Keywords:** dieting, weight, narrative analysis, discourse, life stories, neoliberalism

## Abstract

Women are disproportionately associated with the body and interpellated into discourses around the ‘slender ideal’. Many spend years ‘dieting’ to attain and maintain this ideal. Using a feminist-informed narrative psychological approach, this study explores how one woman makes sense of her weight-watching experiences within her life story and how she constructs and negotiates identities. The participant, recruited through a word-of-mouth strategy, is a British white, working-class 64-year-old woman, who shared her lifelong dieting journey through an unstructured interview. Narrative analysis revealed themes of fluctuating control over body weight, mirroring particular events in her life and the tension between chasing the weight loss dream versus embracing bodily acceptance. Findings are discussed in relation to broader discourses and power structures, emphasising their intersection with the specificities of one woman’s life and the psychological toll of sustained weight consciousness. Merits of a life story and narrative approach are also highlighted.

## Introduction

In today’s postmodern neoliberal societies of industrialised nations, we are all held responsible for our lives, including our health (Overend et al., 2020) and are engaged in identity projects, which entail not only who we are to ourselves, but our perceived (moral) worth in society (Rose, 1998). We are continuously engaged in performing and thus constructing and (re)positioning ‘our selves’, as we live in dynamic social environments and changing (discursive) contexts. We constantly have to position ourselves – our identity, values and worth – anew (Keupp et al., 2013). While this brings a chance of renewal and fresh starts, rather than being restricted by a stable positioning, it also means that our identities, as well as our worth, are precarious – we have to reinvent and prove ourselves continuously, as entrepreneurial subjects (Scharff, 2016).

Our ‘worth’ is frequently linked to our bodies and health (Brown, 2013), with an expectation of continuous efforts for self-optimisation (Nehring and Röcke, 2024). For women, their value is disproportionately linked with their bodies, with a slim embodiment seen as the ideal of femininity (Lupton, 2018). In recent years, these ‘ideal slim’ bodies are also allowed some muscles, and a few (well-toned) curves, as these are signifiers of a well-maintained and allegedly healthy body, but the overall ideal look is still that of slimness with no surplus ‘flesh’ (Ferguson et al., 2021). Despite the critique of these unrealistic expectations by many in academia (Johnston and Foster, 2025; Lupton, 2018; Malson et al., 2023; Tischner, 2013, to name just a few) and in wider culture (e.g. body positivity and fat acceptance movements) links between slenderness, health, beauty and femininity persist as a dominant and powerful force (Malson et al., 2023; Tischner, 2013).

Of course, it is not news that women have historically been, and still are, associated more with their bodies, emotions and irrationality in contrast to men, who have been associated with the mind, activity, rational thinking etc. (Pavco-Giaccia et al., 2019). While men are also subjected to an increasing level of appearance pressure (Malik et al., 2021), in general, men’s bodies are still just *one* of the factors that make up their ‘mosaic masculinities’ (Wagner, 2016). In contrast, a woman’s body and appearance constitute near enough monolithic signifiers of women’s worth (e.g. Tischner, 2013). This worth is further tarnished by women’s bodies having a long history of being constructed as uncontrollable and unruly, and insufficiently contained and controlled (Lupton, 2018) . The leaky female body reflects the unrestrained nature associated with women, together with their being considered more emotional and irrational (Lupton, 2018). In concert with this, women themselves are positioned as needing restraining and controlling, to putatively ensure a functioning society (LeBesco, 2004). Thus, women’s bodies are still essentially monolithic signifiers for who and what they are and capable of, and thus also as signifiers of being ‘other’ to men (e.g. Pavco-Giaccia et al., 2019).

This positioning of women as ‘other’, and less valued through their bodies is compounded in fat women, by the construction of (men and women’s) fat bodies as undisciplined and uncontrolled (Monaghan et al., 2022; Tischner, 2013). The collective knowingness about fat female bodies positions fat women as lazy, stupid (particularly in regards to diet and health) and irresponsible, and thus also as ‘bad’ health citizens, who do not heed the dictum of self-optimisation (Lupton, 2018; Tischner, 2013). Fat women are thus entangled in a discursive health/appearance/femininity nexus which positions them as inferior to slim women, as well as ‘fat’ and slim men, on numerous dimensions. In combination, this positions fat women as abject and their control and constraint as essential for a healthy and successful society.

Being subjected to such discourses and attitudes towards fat women on the one hand, and the idealisation of slim feminine embodiment on the other means that self-stigmatisation is a frequent experience in fat women (Puhl et al., 2020). Self-stigmatisation involves individuals internalising dominant discourses and constructions about fat women as less valuable, less attractive and ultimately less feminine and in return feeling neither desirable nor loveable, even if their own direct experiences do not necessarily reflect this (Gailey and Harjunen, 2019). As such, they may experience romantic relationships as precarious, with the resulting constant fear of being left by their partner.

Considering that fat embodiment is vilified on so many levels, it is unsurprising that weight-loss is assumed as the ideal solution to gain health, as well as (self) worth, professional respect, satisfactory romantic relationships and so forth. (cf. Tischner, 2019). Many attributes that are closely linked to our identities are also associated with our appearance and body weight. The neoliberal ideal that we are continuously improving our selves, and prepared to put in the graft, brings with it the obligation to continuously strive towards our *best* possible self and body (Engeln and Salk, 2016). Weight-loss is one of the technologies of the self that promises to achieve such an optimised self and with it health, good relationships and success (e.g. Tischner, 2019).

As such, we were interested in how these complex dynamics around body weight and identity are negotiated in the narrative accounts of women who identify as being ‘lifelong dieters’. The specific aims were (1) to explore how women make sense of their experiences of ‘weight-watching’, weight loss and weight gain, in the context of their life stories and the discursive resources available to them and (2) to explore how the participants construct and negotiate various identities, as narrated in their life stories. Here we report on one woman’s life story narrative taken from a small collection of life story interviews. This participant’s story was selected for reporting here due to its richness and detail and, importantly, the enthusiastic, vivacious and humorous manner within which it was told.

## Methodology, materials and methods

We chose to employ a narrative psychological approach for the study. Murray (2003: 95) asserts that ‘Narrative psychology is concerned with the structure, content, and function of the stories we tell each other and ourselves in social interaction’, is underpinned by the belief that people are story-telling creatures (Atkinson, 2012), and that narratives help bring a sense of coherence, order and unity to our lives (Crossley, 2000; Ricoeur, 1991). Narrative psychology shares with discursive approaches certain social constructionist assumptions and is similarly concerned with the study of self and identities (Crossley, 2000). However, in contrast to discursive approaches, narrative psychology is premised on the belief that our identities are ‘grounded in “cultural” forms of language and sense-making, whilst still maintaining a sense of the “internal”, “coherent” and “personal” nature of self-experience’ (Crossley, 2000: 533). Similarly, Schiff (2012) argues that whilst narrations are undeniably constructions, they are constructions which say something about aspects of our experiences.

Different ways exist to approach the analysis of a narrative account, ranging from realist and cognitive approaches to a contextualist approach, the latter of which considers the broader social, cultural and historical context within which the account is produced and where it is assumed that aspects of the narrative are shared by other members of the society (Murray, 2003). This is the approach we adopted, whereby we assume that a narrative account will consist of a number of culturally shared discourses, but nevertheless will also tell us something about the ‘personal’ nature of the participant’s experience (Crossley, 2000) and, importantly, how those discourses (e.g. about weight and dieting) ‘hit’ and ‘bruise’ us (Ahmed, 2017: 30; as cited in Thompson et al., 2018).

### Ethics approval

The research was reviewed by the Psychology Local Research Ethics Committee at the first author’s institution and approved 28/02/2019. The participant provided written informed consent prior to take part and for publication.

### Life story research

We decided to employ a life story methodological approach (e.g. Atkinson, 2012) in order to capture the broader context within which the participant’s ‘dieting stories’ could be situated and made sense of. Just as people’s talk and practices do not exist in a social vacuum, life events and occurrences do not take place outside of the context of a whole life lived or, as Bruner (1993: 64) says, ‘People do not deal with the world event by event. . .They frame events. . .in larger structures’. A further advantage of life story interviews is that it places the participant centre stage, allowing them to guide the direction of the interview with minimal ‘interference’ from the researcher (Murray, 2003). This approach aligns well with one of the aims of feminist psychology, namely, attempting to address the researcher/researched power imbalance characteristic of traditional psychological research (Wilkinson, 1988).

Data presented here, from a single participant, comes from an ongoing project seeking to explore women’s narrative accounts of their dieting attempts, weight loss and weight gain throughout their life course. After receiving ethical approval from the first author’s institution, recruitment posters were displayed in numerous outlets across a city in the north of England. Personal contacts and a word-of-mouth strategy were also used in the recruitment process. The participant of concern here contacted the first author through a mutual acquaintance and the interview took place in her own home. Prior to the interview, the participant had received and read a participant information sheet.

Before commencing the interview, the participant was given the opportunity to ask questions and a written consent form was completed. She was assured of confidentiality and that any potentially identifying details would be removed or changed, with the option for her to check over the transcript before analysis began. The participant’s self-chosen pseudonym is ‘Gertie’ and all other names are also anonymised. Gertie is a white, working-class woman from a village in the north of England and at the time of the interview was 64 years old.

The interview took place in April 2019, audio-recorded with permission and lasted 1 hour 8 minutes. Gertie was very forthcoming, animated and enthusiastic throughout the interview, which produced a rich and detailed narrative account. In line with narrative life story research (Murray, 2003) there were only two initial questions, although a small number of questions were asked at appropriate moments during the interview. The initial questions, based around the study’s research aims, were:

I’d like you to start with telling me a bit about yourself and a summary of your life to date. For example, where you were born and raised, your family background, and things you have done between then and now.

I’d now like you to tell me about your earliest memories of becoming aware of weight, and perhaps any attempts to control your weight, and how those experiences continued from then until now. In addition, if there have been any times or events in your life when your weight has become more or less important to you.

### Data analysis

The steps outlined by Crossley (2007) were broadly followed to conduct the analysis. In addition, our readings were also guided by several narrative analytic questions, namely: how do aspects of the story position Gertie and other actors? Whose interests are served by different elements of the narrative? What are the key turning points in the narrative and points of tension? How did the interviewer’s presence, interactions, identity and perceived views on ‘dieting’ likely shape Gertie’s story? Following transcription, we read the transcript multiple times and noted in the margin any initial thoughts about themes or patterns of meaning woven through the narrative, as well as notes pertaining to our analytic questions. Second, we re-read the transcript whilst listening to the audio-recording and made notes about the various narrative tones (i.e. the *way* in which the story was told) adopted during the narrative. For example, angry, resentful, hurt and humorous tones were among the many that were noted. Third, we went through the transcript making more detailed memos than those made previously related to possible themes, whilst also identifying use of imagery and stock phrases. For example, memos such as ‘weight loss seems illogical’ and the ‘cyclical nature of weight loss goals’, and imagery such as ‘I’d blown up like a huge balloon’. Following this, we reviewed all the memos to identify those with shared meanings and grouped these together to produce preliminary themes. For example, many memos pertained to significant weight fluctuations, and these were commonly stitched into stories about contemporaneous, significant events and turning points in her life. These formed one of the themes, which relates to the lack of control that Gertie implied she had over her body weight and simultaneously various events in her life. Fourth, we examined aspects of the data wherein various identities were constructed. For example, ‘the lucky wife’, ‘the girl made good’ and ‘Peter Pan’. Finally, we considered the narrative as a whole and wove together the themes in order to tell the story of Gertie’s decades of ‘weight watching’.

### Overview of Gertie’s life story

At the time of the interview, Gertie was 64 and retired. She was raised in a village in Yorkshire where she still lives with her second husband. At 29, she married her first husband and at the age of 30 had her first and only child. Gertie reported gaining six stones in weight during her pregnancy, having previously been ‘very skinny’. The birth was medically complicated and required several months of recovery. When her daughter was two, the family geographically relocated for her husband’s job. Three years later, Gertie discovered her husband was having an affair. The couple attended marriage counselling and when asked about his infidelity, her husband blamed it on Gertie for her weight gain. Gertie characterised this as being a ‘trigger point’. She stopped eating and ‘lived off coffee and fags’ which saw her weight plummet to six and a half stone. Six months following counselling, she left her husband and returned to Yorkshire with her daughter.

During the following few years, Gertie reported slowly regaining weight, going up to eleven and a half stone, which she described as ‘a decent weight’. When her daughter was 10, Gertie met her current husband with whom she has been for the last 25 years. During those years, Gertie’s weight has fluctuated significantly, and she has tried many different diets. Her husband is 9 years her junior and this is a source of anxiety for Gertie, concerned that he may no longer find her attractive. Gertie’s narrative was overwhelmingly characterised by a sense of conflict; on the one hand, she recognised the futility of chasing the elusive weight loss dream but on the other hand, she struggled to accept and embrace her body size and weight.

## Findings

Three narrative themes were identified during the analytic process, two of which are presented here. The first pertains to the dramatic fluctuations in Gertie’s weight during her adult life, but such fluctuations were embedded in the context of other events in her life, many of which were unexpected, traumatic and out of her control. The second theme relates to the puzzle of weight loss, with Gertie searching throughout her adult life for the ‘magical’ weight loss solution. Yet, her weight loss ambitions were interjected with self-questioning about whether she should relinquish the weight loss dream and embrace self-acceptance.

### Transcription notation

. . . pause/hesitation

( . . . ) text omitted due to word constraints

M: (first author, interviewer)

***From ‘unusually skinny’ to ‘a huge balloon’: Unruly bodies, unruly lives***.

Near the beginning of the interview, Gertie talked about her first marriage at the age of 29:

G: errm. . .ooh I would say (. . .) up until being married I was very skinny

G: (. . .) well, skinny for me, you know ‘cos if I’m sort of round about the. . . (. . .) 9 stone mark then I do look skinny

M: hmm

G: you know. . .unusually skinny. . . (. . .) I was always 9 stone 2. . .always (. . .)

G: when I was pregnant basically I put on an awful lot of weight, I mean I went up to 15 stone ( . . . ). . .I’d blown up like a huge balloon

Gertie’s account in the above extracts was framed as a type of ‘before and after’, transformational narrative. The pre-marriage, pre-pregnant body is described as ‘very skinny’ and ‘unusually skinny’, evoking a powerful image of a fairly extreme form of embodiment. Gertie didn’t comment on how she *felt* about being ‘skinny’ but her emphasising this suggests it was of significance to her (and to the unfolding ‘weight’ story) and possibly part of her identity (i.e. a skinny woman), something to which we return later. Her pregnancy is marked as a turning point in her weight history narrative when she states ‘Oh yeah when I was pregnant basically I put on an awful lot of weight’, and the use of evocative imagery by describing herself as having ‘blown up like a huge balloon’; this provides a stark contrast to the earlier description of her pre-pregnant self as ‘unusually skinny’. Gertie’s account of her pregnancy could be regarded as the genesis of her subsequent weight gain (see Williams, 1984), a rhetorical device serving as an anchor point for the rest of her story which was not only characterised by ‘weight cycling’ but also numerous unexpected life events, some of which were traumatic and out of her control. One such event was the birth of her daughter:

G: basically I had a very difficult birth. . .erm. . .Rachel [daughter] unfortunately died and erm

M: oh God

G: and had to be revived. . .I mean she’s alive

G: and she’s fine, she’s 35 and living in Australia, has the life of Riley and she loves it you know. . .erm she’s a vet and she. . . and er she, amongst other things that she does, ‘cos she works as a vet and works as a waitress, probably to keep up the lifestyle out there, you know ‘cos she lives in Sydney (. . .)

The birth of Gertie’s daughter was ‘very difficult’ and the baby is initially reported here, arguably for dramatic effect, as having ‘unfortunately died’. This was a shocking moment in the interview, prompting the interviewer’s response of ‘Oh God’. Gertie then makes a temporal leap to the present, perhaps to reassure the interviewer that there is a happy ending to the story – not only is her daughter alive but she is also the ‘girl done good’, by constructing her daughter as leading a happy and successful life in Australia. Gertie then returns to the original story of the link between her weight and pregnancy:

G: when I came out of hospital, that’s when I started to be aware of my weight, because obviously, they were saying ‘you’re getting so big, you’re getting so big’ and I was really big and people were saying ‘I can’t believe you’ve gone, you know, in 9 months you’ve put on. . .’ (. . .) what was it. . .6 stone? Erm (. . .) so when I had Rachel. . .obviously I didn’t lose the weight straight. . .well I didn’t lose the weight, end of. . .erm, ah, I think I’d got down to about eleven and a half stone after I’d had her

Following discharge from hospital, Gertie characterises that time as another turning point, a moment of awareness of her weight, something that she attributes to other people telling her she was getting ‘so big’. She then discursively confirms the truth of what ‘they’ were saying in stating ‘I was really big’, thereby closing down opportunity to challenge the gendered and culturally devalued representation of her body. Gertie then draws on the familiar post-partum ‘getting your body back’ narrative (Neiterman and Fox, 2017) but situates herself as the mother who failed to do so: ‘well I didn’t lose the weight, end of’. She then continues to provide vivid details of the traumatic health complications she experienced during the weeks and months following the birth of her daughter, including having ‘a complete blood transfusion’ and ‘a hundred and forty-four internal stitches. . .and they all came away in septic boils’. This detailed and graphic account of the ‘difficult birth’ event arguably functions as a means of justifying why she ‘failed’ to lose the weight gained during pregnancy. Needing to account for her ‘failure’ is unsurprising given the cultural pressures exerted on post-partum women to achieve their former pre-pregnancy weight and shape (Neiterman and Fox, 2017).

Gertie ties up this chapter in her life with the following: -

G: (. . .) for six months afterwards I didn’t feel as if my head was attached to my body. . .so it was probably the next Spring, by the time she [daughter] was. . .you know. . .a good six to eight months old, then I started to feel. . .and. . .that I was coming back to normal

Here, Gertie emphasises the impact of the experience on her, not only physically but also existentially, drawing on the metaphor of the head feeling detached from the body. The ‘difficult birth’ story is then concluded by stating that when her daughter was ‘six to eight months old, then I started to feel. . .and. . .that I was coming back to normal’. Thus, an approximation of normality had been restored in terms of her physical health at least; however, much of the weight gained during pregnancy hadn’t been lost, an aspect of the narrative which Gertie re-introduces in the telling of the next chapter of her life. When her daughter was two, the family moved to a different part of the country for her husband’s work. Shortly after, she discovered that her husband was having an affair:

G: so consequently that upset me. . .erm. . .we went to see a marriage guidance counsellor and I think in some ways this might have been a trigger point because we. . .we sat there and the marriage guidance counsellor said to him, you know, ‘wha. . .what, why. . .why, can you think of why?’ and he went ‘she’s thirty-five, she dresses like a frump’ (. . .) ‘and she. . .she’s put on so much weight, it’s unbelievable. . .she’s not trendy, we don’t go out anymore’ and of course, I blew my top and said ‘yes, I’m a thirty-five year old woman with. . .with a five year old child that I’m trying to take care of’. . .but. . .but the thing about the weight and dress. . .stuck in my mind

G: I think. . .and that’s. . .and. . .at the time I smoked. . .and I just stopped eating

G: I just stopped. . .I lived off coffee and fags. . .and long story short, it went on for another six months. . .er I decided I left [name of place] and I came home. . .I left him there with her [mistress]. . .right?

G: by the time I got home in 19. . . (. . .) I’d be thirty-six coming up thirty-seven. . .right, so Rachel was six coming up seven, I was down at six and a half stone

G: I was just so thin

In these extracts, Gertie talks about a ‘trigger point’ in her weight life story when, at counselling her husband blames his affair on Gertie’s weight gain, dressing ‘like a frump’ and not being ‘trendy’ and thus drawing on a discourse of ‘heterosexual male infidelity’ which is constructed through a number of interlinked assumptions about the (‘natural’) male sex drive (Hollway and Jefferson, 1998), male entitlement to women’s bodies and the expectation that women put male needs and those of others before and above their own (Bordo, 2003). This discourse functions to shift the blame and responsibility on to women when ‘things go wrong’ in the relationship (and in this case, serves to blame Gertie for his infidelity). Gertie depicts her reaction as being one of anger, using the metaphor ‘I blew my top’ but nevertheless acknowledges that his remarks had a long-lasting impact: [it] ‘stuck in my mind’. Gertie constructs this remark as being the trigger for her stopping eating and dropping her weight to six and a half stone. This could be interpreted as an act of ‘proving him wrong’, albeit an extreme and self-destructive reaction. She also left her husband during this period, a point in the story where she becomes the character with agency (McAdams and McLean, 2013), her leaving being an act of resistance to not only her husband’s infidelity but also his blaming the affair on Gertie

### The promised land of weight loss. How to get there?

A few years after leaving her first husband, Gertie met Steven, her current husband. She characterises the 10 years after meeting him as a period of weight stability: ‘I was fine. . .absolutely fine’. From her weight plummeting to six and a half stone, she reports gradually regaining weight (weighing eleven and a half stone), achieving ‘a decent size’ but noting she was still ‘conscious of size’ and had ‘been on various diets in between time because I’d noticed it climb, climb, climb’. Throughout the interview, Gertie refers to the puzzle of weight loss and seems to be continually searching for the ‘holy grail’ but, at times, appears conflicted over whether she should continue in her dieting efforts or accept herself as she is, and be thankful for the life she has. Below, Gertie narrates her experiences of weight loss attempts and the sense of bewilderment this produces: -

G: so yes, I’ve been to Slimming World and I’ve lost a stone, and I’ve been to Weight Watchers and lost a stone but then. . .I go one week and I think I’ve been really, really good and I haven’t lost anything and I think. . .‘oh well stuff that then’

G: and then I start eating again, and then I put on probably more than I did in the first place. . .I mean, funnily enough, I mean I do go to the gym. . .[ . . . ] and Aquafit twice a week. . .erm. . .I had two dogs here, looking after them for sixteen days, walking them four times a day. . .I lost half a stone without even trying

Here, Gertie talks about her dieting efforts and, whilst managing to lose a stone on each of these occasions, she draws attention to times when she hasn’t lost any weight despite having ‘been really, really good’ and the emotional reaction this provokes in her, which is one of rebellion and a sense of futility (‘Oh well stuff that then’). Gertie is drawing on a common and powerful moral discourse around eating practices which functions to categorise foods into ‘good’ and ‘bad’ (Scrinis, 2015) and by way of extension, those who eat ‘good’ foods as good, moral people who exercise self-restraint and control and those who eat ‘bad’ foods as lacking in all such qualities and are therefore morally suspect (Lupton, 2018). Notable here is Gertie’s sense of confusion, conflict and frustration resulting from following ‘slimming advice’ but not achieving the promised results yet having to deny herself the pleasures of eating without restraint. She narrates this in a humorous manner, possibly as she recognises the futility of continually going on diets (based on her own experiences) yet is aware of (and knows that the interviewer is aware of) her persistence with chasing the weight loss dream.

In the second utterance above, Gertie relays how she starts ‘eating again’ and gains more weight than she originally lost. This draws on the well-known ‘weight cycling’ discourse which refers to the repeated weight fluctuations commonly experienced by ‘chronic dieters’ and is said to adversely impact on health (Montani et al., 2015). Gertie is trying to make sense of how, when and why she loses weight, emphasising that she goes to the gym in her weight loss efforts, but when she undertakes activities without the intention of weight loss, she can lose half a stone. Again, this is expressed humorously as (arguably) a means of conveying the illogicality of ‘official’ weight loss advice (e.g. ‘eat less, move more’ – NHS, 2023).

Gertie’s confusion and sense of conflict is further conveyed when she talks about her daughter’s forthcoming wedding:

G: I mean my daughter’s getting married next year and I’m thinking I seriously need to lose weight. . .right? To look. . .you know, like the other Mum. . .the other Mum looks fabulous. . .you know, what’s this blob gonna look like at her wedding? Do you know what I mean, and that goes through your mind. . .I think, ok, ok I can do it, I can do it and every New Year I think I can do it. . .I then I get to sort of like. . .going on holiday and we go on a cruise and I think ‘Do you know what? I’ve paid for that, I’m eating it!’ [laughs]

G: Do you know what I mean? [laughs] it’s really. . .it’s really sickening really but. . .[laughing] it’s stupid and yet. . .and yet other times. . .I’ll sit there and I think ‘Do you know what? I’m not eating that ‘cos it’s not good for me’ It’s. . .I am really a flibberty jib

G: I’ve probably lost thousands through being. . .paying to go to things. . .I mean. . .yeah. . .and I think sixty. . .again, sixty-five. . .why the bloody hell. . .I’ve got to sixty-five. . .I’m not. . .what difference is it going to make?

In the above, Gertie expresses a conflicted sentiment when she talks of her daughter’s forthcoming wedding, which she presents as the next big weight loss goal to look ‘fabulous’ like ‘the other Mum’. Sobal et al. (1999) note that weddings are a particular cultural site where (heterosexual) women (especially ‘the bride’) are the focus of attention and most ‘brides-to-be’ experience a heightened concern about their weight and engage in weight loss attempts prior to the wedding. Such ‘pressures’ may also be extended to other significant female relatives of the bride and groom (e.g. ‘mother-of-the-bride’), as articulated by Gertie here. It is ‘the other Mum’ whom Gertie positions as the figure of comparison and her rival in the weight/beauty stakes; this is unsurprising given that women’s bodies are always the subject of one another’s gaze and as a site of comparison and competition (Guendouzi, 2004). She draws on vivid imagery to describe herself as a ‘blob’, which sits in stark contrast with the prior description of her pre-pregnant self as ‘very skinny’. She talks about how ‘every New Year’ she is filled with renewed hope and optimism believing that she ‘can do it’ [lose weight], envisioning a future ‘slim’ self. However, this sits in contrast to occasions (e.g. on a cruise) when she just wants to enjoy the pleasures of eating. So again, Gertie narrates the tension she experiences between the desire for weight loss and the pleasure derived from food. This tension between restraint and pleasure is narrated as part of her identity where she refers to herself as ‘a flibberty jib’. This is a regional colloquial term used to describe someone (originally women) who ‘flips’ from one thing to another (Griffiths, 2006) and a self-description Gertie uses on several occasions to express what she sees as her lack of consistency in her mission to lose weight.

In the final utterance above, she refers to the ‘thousands’ she has spent on this mission and reflects on the futility of it all, drawing on a discourse of ageing (‘I’ve got to sixty-five. . .I’m not. . .what difference is it going to make?’). In an examination of the narratives of ‘older’ women dieters, Gimlin (2007) found that participants drew on a discourse of ageing which served as a form of resistance to, and a protection from a discourse of dieting ‘to be heterosexually attractive’. Here we can see that Gertie also toys with this discourse momentarily but fails to consistently sustain her investment in it, more frequently drawing on a discourse around the imperative to lose weight and maintain male interest. This is perhaps unsurprising, however, given Gertie’s first husband blaming his infidelity on her weight and her current husband being 9 years her junior. During the interview, Gertie referred to her current husband as ‘Peter Pan’ and emphasised how young he looks for his age. This was a source of concern for Gertie, even though she emphasised several times during the interview that he had never commented on her weight (in fact, he was critical of her dieting). Nevertheless, she often attributed her weight loss attempts as motivated by her desire to maintain his sexual interest. Thus, Gertie’s particular life history (e.g. the behaviour and comments of her ex-husband) means that the ‘let yourself go, lose your man’ narrative may be one within which Gertie is particularly interpellated.

Throughout the interview, Gertie makes reference to the various diets she has tried, and the different types of advice she has been given. As the following extracts illustrate, standard weight loss advice (according to Gertie) hasn’t ‘worked’ and has added to her confusion and ambivalence about the pursuit of weight loss:

G: yeah. . .yeah [laughs] the only way I think I diet [successfully] is if I start eating salad. . .all day every day. . .I mean even when I’ve been on diets before and I ate fruit and veg, they’ve said to me ‘you’re eating too much fruit. . .you’ve got too much sugar going into your body’

G: so how do I win?. . .so the only way. . .I’ve ever lost weight is by basically having salad 24/7. . .every meal

M: and how do you feel when you’re doing that?

G: Shit! [. . .] especially when you’re cooking a meal for a husband and he’s going like ‘You can’t sustain this Gertie for God’s sake. . .you know, it’s healthy eating’ Well sorry Steve but for me it isn’t healthy eating because whatever I eat. . .this is the weight that is, do you know what I mean? I think I almost. . .like stages in my life, I’ve been certain weights like nine stone two, eleven eleven, thirteen and a half stone I’m probably a bit overweight now, you know, ‘cos as I say I’ve just lost half a stone, I’ve probably just tipped. . .what am I now. . .I think I’m thirteen. . .thirteen ten or eleven something like that. . .which in my mind I’m thinking ‘if I were thirteen I’d be happier’. . .do you know what I mean?

Adopting a tone of frustration, Gertie claims that the only way she has lost weight in the past is to eat ‘salad. . .all day everyday’ and has been admonished for ‘eating too much fruit’, which seems counter to the dominant ‘healthy eating’ advice of eating at least five portions of fruit and vegetables a day (e.g. NHS, 2022). This appears to be a source of utter frustration to Gertie as she knows that only eating salads isn’t sustainable and makes her feel ‘shit!’. Added to this, whilst only eating salads herself, she cooks meals for her husband, thus drawing on a gendered discourse of provision and restriction – that is, women providing food for men whilst restricting their own consumption. The provisioning of food for family members is intimately bound up with constructions of heteronormative femininity and part of the performance of gender (Cronin et al., 2014). Juxtaposed with this though, women are expected to monitor their own food intake in efforts to ‘watch their weight’ to cultivate the idealised heteronormative feminine slender body (Cronin et al., 2014).

## Discussion and concluding remarks

There were two key turning points in Gertie’s narrative in relation to her weight and dieting attempts, something which we discuss further here. The first of these was her pregnancy where she tells of gaining six stones in weight, having previously been ‘very skinny’. Gertie doesn’t say how she felt about being ‘skinny’ but the fact that she mentioned this several times suggests this was of significance to her and to the story. In trying to make sense of Gertie’s subsequent and repeated weight loss attempts following pregnancy and over several decades, (and the struggle to accept her ‘heavier’ self), there are a number of elements to consider.

First, a woman’s body shape and size are commonly part of their self-identity and how others see them (Patel et al., 2005), something unsurprising in societies where a woman’s appearance is a central site of judgement. For Gertie, it is possible that her pre-pregnant ‘skinny’ self was a significant part of her identity, and the subsequent repeated weight-loss attempts were, in part, an expression of a longing for the body/self that once was. However, a change in body weight/size doesn’t just play out in the social and psychological realm of identity; bodies have a material existence and are experienced in our everyday movements and activities (Råheim et al., 2024). In a study exploring experiences of self-directed weight loss after ‘severe obesity’, Råheim et al. (2024) found that participants who had been slim (or not particularly heavy) and active in childhood/adolescence regarded *that* state of embodiment and physicality as their ‘normal’ and, following significant weight loss, felt ‘at home’ in a body size more aligned with that of their younger self. For Gertie, it’s possible that the significant weight gain during and following pregnancy was experienced at a very physical level, a sense of unfamiliarity in the way her body moved etc. This sense of ‘alienation’ from her body may also have contributed to her quest for weight loss.

A second key turning point in Gertie’s story, and pertinent to her repeated dieting, was her first husband’s infidelity and his blaming of this on Gertie’s weight gain, an age-old gendered and patriarchal discourse, one of which provides fertile ground for encouraging women to engage in an array of body management practices (in Gertie’s case, ‘serial dieting’) whereby the body becomes a never-ending project to be worked on (e.g. Elias et al., 2017). Gertie’s reactions to her ex-husband’s comments were two-fold: to virtually stop eating which resulted in dramatic (and health-threatening) weight loss, yet she also left her husband, signifying an act of resistance and agency. These two different reactions could be interpreted as examples of how power structures and discourses ‘hit’ and ‘bruise’ us (Ahmed, 2017: 30; cited in Thompson et al., 2018) (as Gertie said, in relation to her ex-husband’s comments, ‘the thing about the weight and dress. . .stuck in my mind’) whilst simultaneously the injustice of those power imbalances are recognised and resisted (i.e. Gertie’s anger towards her ex-husband and her act of leaving him).

However, Gertie didn’t embrace her dieting attempts without question. She expressed a sense of conflict, caught between desiring enjoyment and pleasure, but also wishing to lose weight. When reflecting on whether she should abandon ‘dieting’, she drew upon a discourse of ageing which potentially positioned her as being absolved from the cultural imperative to achieve and maintain weight loss. Research conducted around 20 years ago also noted similar findings whereby an ageing discourse offered a degree of protection from and served as a means of resistance to the slender body ideal imperative (e.g. Gimlin, 2007). However, it seemed that Gertie struggled to sustain her investment in a discourse of ageing; the ‘promised land of weight loss’ appeared to have a greater pull, suggesting the power and potency of this discourse, as noted in more recent body image research with ‘older’ participants (Markey et al., 2020). Weight loss discourse is likely very much intertwined in Gertie’s life history – her former husband blaming his affair on her weight gain. Her current husband being 9 years her junior, also potentially weakens the protection offered through the ageing discourse. This demonstrates how we are interpellated within some discourses more so than others (i.e. how some discourses ‘speak to us’ more than others), and the meanings people attach to weight, dieting, ageing and so on are shaped by our individual life histories (among other things); this is something which may not have become apparent if the interviews had solely focused on stories of dieting, highlighting the advantages of conducting life story interviews. Furthermore, we argue that the capturing of the psychologically damaging and oppressive nature of patriarchal discourses around women’s bodies and weight (cf. Bordo, 2003; Malson, 2008) is an advantage of adopting a narrative psychological approach in that the participant’s voice remains foregrounded, something which has been the subject of criticism from several critical and feminist authors with regard to purely discursive approaches (e.g. Crossley, 2000; Thompson et al., 2018).

This paper contributes to the substantial body of feminist and critical research and theory that examines the ‘conditions of possibility’ which may serve to produce women’s body management practices (e.g. ways of eating, exercising, dieting etc.) and their related subjectivities (e.g. Bordo, 2003; Tischner, 2013; Woolhouse et al., 2012). Specifically, by adopting a life story approach, we were able to situate stories of dieting within the context of the complexities of one woman’s life events. In addition, deploying narrative analysis and therefore considering the *ways* in which the story was recounted allowed us to capture some of the emotional and psychological impact of structural and discursive forces on the participant. Finally, focusing on the weight-related and embodied experiences of a woman in ‘later’ life provides a valuable contribution to the literature, given that scholarship on female embodiment is highly skewed towards young women, possibly underpinned by the assumption that older women are largely immune from weight and appearance pressures (Gimlin, 2007).

Given the paucity of research on older women’s embodiment and weight-watching practices (Markey et al., 2020), fruitful future avenues may be to conduct longitudinal qualitative research to explore how women make sense of weight, food, eating and body management practices through their life course. This would be of value given that such meanings are open to change, being shaped by differing cultural and historical contexts as well as individual life histories and personal relationships.

## Data Availability

Data can be made available on request but is not available in a public data repository due to the personal nature of a life story interview.*
